# Patient-reported outcome measure comparison of two cemented primary total hip arthroplasty implant combinations for osteoarthritis: a regional New Zealand study

**DOI:** 10.1007/s00590-026-04734-w

**Published:** 2026-04-02

**Authors:** Amy Pearce, Chaitanya Joshi, Georgina Chan, Tony Lamberton, Simon MacLean, Andrew Vane, Kim Hébert-Losier

**Affiliations:** 1 Division of Health, University of Waikato, Hamilton, New Zealand; 2 Centre for Sport Science and Human Performance, Waikato Institute of Technology, Hamilton, New Zealand; 3 School of Public Health, University of Adelaide, Adelaide, Australia; 4 Tauranga Hospital, Tauranga, New Zealand; 5 Grace Orthopaedic Centre, Tauranga, New Zealand; 6 Grace Orthopaedic Centre, Tauranga, New Zealand

**Keywords:** Hip replacement, Polyethylene, Ethnicity, Health-related quality of life, Health disparity

## Abstract

**Purpose:**

Compare patient-reported outcome measures (PROMs) of two cemented implants in primary total hip arthroplasty (THA) for osteoarthritis in a New Zealand regional joint registry. Identify patient predictors of poorer PROMs.

**Methods:**

We analysed observational data from primary THA surgeries for osteoarthritis performed between 1 January 2003 and 30 June 2023, with at least one recorded PROM (*n* = 1365) from a regional joint registry. We compared preoperative, 1-year, 5-year, and 10-year PROMs in the cemented, highly crosslinked polyethylene Exeter^®^ X3 Rimfit cup (Rimfit) and its conventional polyethylene predecessor, the Exeter^®^ Contemporary Flanged cup (ECF) with the same cemented Exeter^®^ V40 stem. We investigated six patient factors and their influence on physical function, and mental and physical health PROMs.

**Results:**

No significant difference in physical function PROMs were noted between implants at any timepoint. Both implant combinations exhibited excellent (> 41) mean postoperative Oxford hip scores based on published thresholds (Rimfit: mean 41.30 ± SD 8.46; ECF: mean 41.64 ± SD 8.67). Mental health was significantly better preoperatively and at 1-year and 5-year with the Rimfit based on Veterans RAND 12-Item Health Survey. Public funding was a significant and clinically meaningful predictor of poorer preoperative outcomes in all PROMs and in both cohorts, followed by comorbidity status. The strongest significant predictor of poorer postoperative PROMs was poorer preoperative PROMs, followed by Māori ethnicity.

**Conclusion:**

Both implant combinations demonstrated similar PROMs up to 10 years, but the Rimfit exhibited superior mental health. Public funding, comorbidity status, and Māori ethnicity were predictors of statistically significant and clinically meaningful poorer preoperative and postoperative PROMs.

**Supplementary Information:**

The online version contains supplementary material available at 10.1007/s00590-026-04734-w.

## Introduction

Total hip arthroplasty (THA) is widely performed, highly successful, and significantly improves quality of life in patients with advanced hip osteoarthritis [1]. While literature has traditionally focused on implant survival and radiographic outcomes [2], there is a growing emphasis on patient-reported outcome measures (PROMs) as critical success indicators of hip arthroplasty [3, 4]. When new or upgraded implants are introduced, they are often assumed to perform at least as well as their predecessors, and studies rightly focus on survivorship. However, it remains to be determined whether they offer comparable patient-reported clinical benefit over time, despite survival outcomes.

The Exeter^®^ Contemporary Flanged cup (ECF) was the most popular cemented cup in New Zealand (NZ) until the introduction of the Exeter^®^ X3 Rimfit cup (Rimfit) in 2010, which was intended to improve wear [5] with the introduction of highly-crosslinked polyethylene. A single centre study (*n* = 108) reported excellent (> 41) [6] 10-year ECF Oxford hip scores, with a median OHS of 43 out of 48 [7]; and a 15-year NZ regional joint registry study on the ECF (*n* = 263) reported a good (34–41) [6] mean OHS score of 39.5 ± 9.58 [8] (mean ± SD).The Rimfit has also performed well in studies, exhibiting a good mean OHS [6] of 38.5 ± 7.9 (*n* = 178) at 6 months and an excellent [6] score of 42.4 ± 6.2 (*n* = 44) at 5 years in a NZ regional joint registry study in elderly patients [9].

Our recent study identified no evidence of significant differences in survival, or reasons for revision between these two cemented implants [10], and while van Winterswijk et al. [11] identify good-to-excellent OHS outcomes based on published thresholds [12], in both implants, comparison only compared 6 months and 5 years (*n* = 261). No difference in short- and medium-term PROMs was noted, yet no predictors of outcomes or long-term outcomes were investigated. To address this gap, we compared PROMs up to 10 years between the implants using a relatively large NZ joint registry dataset (*n* = 1339) and investigated the influence of six patient characteristics on PROM outcomes.

## Materials and methods

### Data source and collection

Anonymised, prospectively collected PROM data of all cemented primary THAs for osteoarthritis performed between 1 January 2003 and 30 June 2023 (ECF 2003–2013, Rimfit 2010–2023) in the Bay of Plenty region of NZ were retrieved in January 2024 from the Tauranga Orthopaedic Research (TOR) regional joint registry. TOR is a non-profit organisation that maintains a registry of all Bay of Plenty joint arthroplasty surgeries separate from the NZ National Joint registry. With consent, patient data are entered into SOCRATES software (version 3.5.8.26 10150). The OHS, Western Ontario and McMaster Universities Osteoarthritis Index (WOMAC), and Veterans RAND 12-Item Health Survey (VR-12) physical and mental scores are routinely collected preoperatively and at 1-year, 5-year, and every 5 years thereafter. The University of Waikato’s Human Research Ethics Committee (HREC2023#12) granted ethical approval for this study. All analyses were conducted in RStudio (Vienna, Austria) [13], with methodological oversight from a specialist in public health and biostatistics. Observations were eligible for analysis when at least one PROM was recorded for patients who underwent a primary THA for osteoarthritis with a cemented Exeter^®^ V40 stem and either a highly crosslinked polyethylene Exeter^®^ X3 Rimfit cup or an Exeter^®^ Contemporary Flanged cup (Stryker Orthopaedics, Mahwah, NJ) with conventional polyethylene. Follow-up windows were necessarily broad due to the registry-based nature of PROM collection; however, given the stability of PROMs beyond 12 months following THA, variation in response timing within each window is unlikely to have materially influenced outcomes. This study was reported following the Strengthening the Reporting of Observational Studies (STROBE) guidelines [14].

### Patient-reported outcome measures

Preoperative PROM data comprised scores recorded prior to surgery. Follow-up data were defined as: 1-year (10–14 months postoperatively), 5-year (4–6 years postoperatively), and 10-year (9–11 years postoperatively). To aid interpretation of differences between implant cohorts and patient subgroups (sex, age, body mass index [BMI], American Society of Anesthesiologists [ASA] rating, funding, and ethnicity), we applied minimal clinically important difference (MCID) thresholds derived from distribution-based estimates [15] using the preoperative scores from each cohort. Although anchor-based MCIDs for OHS and WOMAC have been published, MCID values are known to be population and context dependent. Given differences in population characteristics, health system structure, and baseline PROM distributions, externally derived MCIDs may not be directly applicable to the New Zealand arthroplasty population. In addition, anchor-based methods often rely on patient satisfaction measures not available in registry data. We therefore used a distribution-based approach (0.5 SD of baseline scores) to derive internally consistent MCID thresholds relevant to the study population. While MCID is primarily designed to assess within-patient changes over time [16], it can offer contextual insight into whether observed, between-group differences are likely to be clinically meaningful [17]. A clinically meaningful difference in score was deemed one that exceeded our established MCID thresholds. For patients undergoing bilateral THA during the study period, PROMs were collected and recorded separately for each hip and corresponded to the specific surgical episode.

### Patient characteristics

Patient characteristics included sex (male, female), ethnicity (NZ European, NZ Māori, Other), ASA rating (1, 2, 3, 4), and funding source (public, private) treated as categorical or rank variables, with age and BMI treated as continuous variables. “Other” ethnicity represented a small, heterogeneous subgroup (*n* = 5) and was thus removed from further analysis. The small number of ASA 4 observations (*n* = 10) were converted to ASA 3 for analysis. Funding source was included as a marker of healthcare access pathway. While funding source may be associated with socioeconomic factors, it does not capture the multidimensional nature of socioeconomic status and was not interpreted as a direct surrogate for socioeconomic status.

### Statistical analysis

Baseline categorical (sex, ethnicity, ASA, funding) and mean continuous (age, BMI) patient characteristics; and preoperative, 1-year, 5-year, and 10-year OHS, WOMAC, VR-12 physical and mental health scores were determined for each component. Missing data were handled using pairwise deletion, meaning that all available observations were used for each analysis without requiring complete data across all variables. Missing data may be due to incomplete questionnaires, non-returned questionnaires, or study withdrawal. Because implant use was strongly structured by calendar time, with the predecessor implant predominantly used between 2003 and 2013 and the successor implant between 2010 and 2023, the two cohorts were not exchangeable in time. As a result, implant group was highly correlated with era-related factors including patient selection, perioperative care, rehabilitation pathways, registry completeness, and PROM collection practices. To avoid conflating implant effects with secular trends, we did not perform a pooled regression with implant as a single exposure across eras. Instead, analyses were conducted within each implant era to evaluate outcome trajectories and prognostic factors in the clinical context in which each implant was used. We compared categorical variable proportions between implants using Chi-squared and Fisher’s exact tests. Mean age, BMI, and preoperative, 1-year, 5-year, and 10-year PROMs between implants were compared using independent Student’s or Welch’s *t*-tests in presence of unequal variance. Because not all patients had both preoperative and postoperative scores available, comparisons between preoperative and postoperative means were conducted using independent samples *t*-tests rather than paired analyses. Mean preoperative, 1-year, 5-year, and 10-year PROMs within each cohort were compared using ANOVA and post-hoc Tukey. Postoperative observations with OHS < 27 categorised as “poor” [6] were extracted to investigate distinct patient characteristics.

To determine influential patient characteristics on preoperative and postoperative PROMs in each cohort, we performed multiple linear regression with post-hoc residual analysis, influence statistics, variance inflation factor, and Durbin-Watson. Preoperative scores were included as predictors of postoperative scores and thus significant predictors of preoperative scores were removed from postoperative analysis to prevent confounding. Statistical significance was set at *p* < 0.05 with 95% confidence intervals (CI) derived for all testing.

## Results

A total of 1630 (Rimfit *n* = 1267; ECF *n* = 363) primary THAs met the inclusion criteria. Follow- up occurred up to a period of 10 years. Of these, 1365 patients (84%) (Rimfit *n* = 1102; ECF *n* = 263) had at least one completed PROM and were included in the analyses (Table 1). Due to variable follow-up completion and missing covariate data, the number of patients included differed between analyses and time points; the sample size for each analysis is reported in the corresponding tables. Assumptions of multiple linear regression were checked and met. Normality was evaluated visually using Q-Q plots of residuals. Initial models using untransformed outcome data in the Rimfit cohort’s postoperative OHS and WOMAC PROMs showed deviations from normality in the residuals. However, the analysis was not intended for prediction, but rather to examine associations between patient variables and PROMs. Results are considered robust despite this noted departure from normality. The large sample size improves the precision of the estimates and statistical power but does not eliminate the potential for residual confounding or other sources of bias inherent in observational registry data.

There was a significantly higher proportion of ASA 2 (*p* = 0.004), ASA3 (*p* = 0.007), and publicly funded patients (*p* < 0.001) in the Rimfit than the ECF cohort. No significant difference in mean age or BMI between cohorts or between any of their six subgroups (sex, age, BMI, ethnicity, funding, or ASA rating) was noted, except for where the ECF mean BMI was statistically higher in ASA 3 patients (*p* = 0.031).

**Table 1:** Patient characteristics between implant comparison

	Both	Rimfit	ECF	*P*- value
Total, *n* (%)	1365 (100%)	1102 (80.73%)	263 (19.27%)	–
Age (mean ± SD)	71.39 ± 8.60	71.39 ± 8.52	71.38 ± 8.67	0.991
BMI (mean ± SD)	28.00 ± 4.85	28.00 ± 4.93	28.01 ± 4.75	0.970
Sex, n (%)^‡^				
Male	543 (39.78%)	423 (38.38%)	120 (45.63%)	0.031
Female	822 (60.02%)	679 (61.62%)	143 (54.37)	0.031
ASA, n (%)^‡^				
1	195 (14.52%)	146 (13.46%)	49 (18.63%)	0.041*
2	824 (60.37%)	660 (60.88%)	164 (62.36%)	0.401
3 and 4	324 (24.12%)	278 (25.65%)	46 (17.49%)	0.007*
Missing	22	18	4	–
Funding, n (%)^‡^				
Public	588 (43.17%)	501 (45.59%)	87 (33.08%)	< 0.001*
Private	774 (57.63%)	598 (54.41%)	176 (66.92%)	< 0.001*
Missing	3	3	-	–
Ethnicity, n (%)^‡^				
NZEU	1258 (94.80%)	1016 (94.43%)	242 (92.02%)	0.102
Māori	64 (4.82%)	55 (5.11%)	9 (3.42%)	0.315
Other	5 (0.38%)	5 (0.46%)	0	0.180
Missing	26	26	0	–

ASA, American Society of Anesthesiologists grading; BMI, Body mass index; ECF, Exeter Contemporary Flanged cup; NZEU, New Zealand European; Rimfit, Exeter X3 Rimfit cup

^‡^Values represent the number and percentage of males and females, ASA 1, 2, 3 and 4, public and private funding, and NZ European, Māori and other ethnicity within each cohort

^*^Significance set at *p* < 0.05

Missing values have not been included in proportion calculations

### PROMs comparison

PROM comparisons between implants can be found in Table 2. No significant difference in mean preoperative, 1-year, 5-year, or 10-year OHS, WOMAC, or VR-12 physical function scores between implants was noted. VR-12 mental scores were significantly higher preoperatively (2.5 points, *p* = 0.005), at 1-year (1.4 points, *p* = 0.042), and at 5-year (2.1 points, *p* = 0.004) in the Rimfit. Within implants, there was a significant time effect across all PROMs examined (*p* < 0.001). Post-hoc Tukey revealed significant improvements from baseline PROMs compared to 1, 5, and 10-years (*p* < 0.001). There were no statistically significant differences in mean PROMs within implant cohorts between 1, 5, and 10 years, except where the Oxford 5-year score was significantly better than the 1-year score in the Rimfit, supporting limited divergence in longer-term outcomes. Given the limited sample size at 10 years and lack of significant difference between 1, 5, and 10-year PROMs, we used 1-year PROMs as the postoperative score for further comparisons due to its substantial sample size.

### Poor postoperative scores

There were 67 patients with poor postoperative OHS (< 27) [6] scores (*n* = 60, Rimfit *n* = 53, 6.3%; ECF *n* = 7, 3.4%). There was no significant difference in patient characteristic proportions of poor postoperative scores, mean age (ECF 74.9 ± 10.1; Rimfit 71.6 ± 8.3 years, *p* = 0.566) or BMI (ECF 26.6 ± 3.6 kg/m^2^; Rimfit 29.3 ± 4.7 kg/m^2^, *p* = 0.106), ethnic groups (*p* = 0.580), ASA ratings (*p* = 0.217), or funding (*p* = 0.684) between the cohorts. The small number of observations in the ECF cohort made it challenging to analyse in further detail (Supplementary Table 1).

**Table 2:** Preoperative and postoperative PROMs comparison

PROM	Rimfit					ECF					*p*-value^¶^
	*n*	Mean	SD	Diff [95% CI]^†^	MCID^‡^	*n*	Mean	SD	Diff [95% CI]^†^	MCID^‡^	
Oxford (0–48)											
Pre-op	837	18.23	± 8.96		4.48	263	17.75	± 8.73		4.37	0.446
Post-op 1-year	842	41.29	± 7.42	23.06 [22.25, 23.82]		238	41.30	± 7.06	22.43 [21.06, 23.81]		0.985
Post-op 5-year	490	42.88	± 6.85			180	41.62	± 8.46			0.074
Post-op 10-year	124	41.64	± 8.67			139	41.30	± 9.58			0.763
WOMAC total (0–96)											
Pre-op	827	54.23	± 18.51		9.26	262	54.82	± 19.27		9.64	0.663
Post-op 1-year	810	14.12	± 14.09	40.15 [38.60, 41.71]		235	12.36	± 12.68	40.40 [37.65, 43.15]		0.069
Post-op 5-year	483	12.41	± 14.29			179	13.18	± 14.47			0.540
Post-op 10-year	124	15.38	± 17.23			137	14.66	± 16.32			0.730
VR-12 physical (x̄ = 50)											
Pre-op	836	27.61	± 8.21		4.11	263	26.52	± 8.22		4.11	0.058
Post-op 1-year	840	44.01	± 10.57	17.05 [16.12, 17.97]		240	43.36	± 10.98	16.37 [14.88, 17.87]		0.405
Post-op 5-year	487	44.43	± 10.37			183	43.55	± 11.76			0.280
Post-op 10-year	125	42.23	± 11.34			138	43.51	± 10.49			0.344
VR-12 mental (x̄ = 50)											
Pre-op	836	46.36	± 12.76		6.38	263	43.82	± 12.85		6.43	0.005*
Post-op 1-year	840	53.31	± 9.40	6.49 [5.49, 7.48]		240	51.91	± 9.41	7.23 [ 5.41, 9.04]		0.042*
Post-op 5-year	487	53.97	± 10.00			183	51.78	± 8.31			0.004*
Post-op 10-year	125	55.16	± 8.86			138	53.87	± 9.26			0.250

ECF, Exeter Contemporary Flanged cup; MCID, Minimal clinically important difference (distribution-based); Pre-op, preoperative; Rimfit, Exeter X3 Rimfit cup; SD, standard deviation; VR-12, Veterans Rand 12-item health survey; WOMAC, Western Ontario and McMaster Universities Index

^†^Difference calculated between the mean preoperative score and the mean 1-year postoperative score

^‡^MCID calculated using the distribution method of 0.5 × SD of the preoperative score

^¶^*p*-value represents the comparison of the Rimfit score to the ECF score for that period

^*^Significance level set at *p* < 0.05

### Linear regression: preoperative PROMs

Funding source was the strongest predictor (i.e., largest coefficient) of baseline PROMs in both cohorts. Publicly funded patients reported substantially worse preoperative scores than privately funded patients, including ~ 8–10 lower OHS, ~ 16–23 higher WOMAC, ~ 5–6 lower VR-12 physical, and ~ 7–14 lower VR-12 mental scores. These differences exceeded our distribution-based MCID across all PROMs. Sex was also a significant predictor, with males scoring ~ 2–3 points better in OHS and VR-12 mental and physical, and ~ 4–8 points better on WOMAC compared to females. BMI showed a small statistically significant effect on preoperative scores (OHS ≈ -0.25; WOMAC ≈ 0.5–0.9) per 1 BMI unit increase, in both cohorts. In the Rimfit, other significant preoperative predictors included higher ASA ratings (i.e., higher comorbidity burden) associated with ~ 2–8 worse WOMAC and ~ 3–6 worse VR-12 physical and mental scores, and Māori ethnicity associated with ~ 6 points lower preoperative VR-12 mental scores compared with NZ Europeans.

### Linear regression: postoperative PROMs

Linear regression for postoperative PROMS was performed using the 1-year postoperative scores for regression due to its larger sample size and lack of significant difference between 1-year, 5-year, and 10- year postoperative scores. Preoperative PROMs consistently exhibited the largest predictive contribution to postoperative scores across PROMs in both cohorts, with each 1-point worse preoperative score associated with a 0.18–0.53-point worse postoperative score depending on the PROM and implant type. In the Rimfit cohort, age at surgery was an additional significant predictor of poorer postoperative OHS, WOMAC, and VR-12 physical scores (≈ -0.1 to − 0.3 per year). In the Rimfit cohort, ethnicity was also a clinically meaningful predictor, with Māori patients having significantly worse postoperative OHS (~ -5 points), WOMAC ( ~ + 6 points), and VR-12 physical (~ − 8 points) scores compared with NZ European. In the Rimfit cohort, BMI was significant only for postoperative VR-12 mental scores, with higher BMI associated with poorer postoperative outcomes. In the ECF cohort, age at surgery predicted worse postoperative VR-12 physical scores (≈ − 0.40 per year). ASA 3 was also associated with worse postoperative WOMAC (7.7 points) and VR-12 physical (5.6 points) scores compared to ASA 1. The R^2^ values (0.08–0.34) from the regression analyses indicate that the included predictors explain only a modest proportion of outcome variance.

## Discussion

A strength of our study is the consistency in key operative factors minimising variation in surgical technique, including the same cemented stem, hospitals, and overlapping surgical personnel. The shared geographic catchment confirms similar underlying demographics, which reinforces cohort comparability.

### Preoperative PROMs comparison

This study addresses an important evidence gap by evaluating the Rimfit’s 10-year suitability as a replacement for the ECF cemented cup. Distribution-based MCID estimates provide a useful benchmark to interpret the clinical meaningfulness of observed PROM changes [16, 18]. The OHS, WOMAC, and VR-12 physical scores all demonstrated substantial and statistically significant improvements from preoperative to postoperative PROMs, far exceeding our MCID thresholds of ~ 4.5, ~ 9, and ~ 6 respectively in both cohorts. Previous research by Yeo et al. [19] indicated improved physical function after THA, with OHS and WOMAC improvements being good indicators of THA success. The VR-12 mental scores in our study showed more modest clinical changes but also exceeded our MCID of ~ 6 and ~ 7 in the Rimfit and ECF, respectively.

### Postoperative PROMs comparison

No statistically significant differences between 1-year, 5-year, and 10-year mean physical postoperative scores within each cohort were noted. All mean postoperative OHS scores exceeded the excellent threshold score of 41 [6]. These findings indicate positive PROM outcomes are achieved using both implants, which are sustained over a 10-year period. This consistency reinforces the implants provide comparable physical outcomes, despite surgeries occurring in two different 10-year intervals. Significantly better preoperative mental health scores in the Rimfit cohort suggest baseline differences in psychological wellbeing between groups that cannot be attributable to implant characteristics. The persistence of higher mental health scores at 1 and 5 years postoperatively is therefore most plausibly explained by the significant influence of preoperative outcomes on postoperative outcomes, rather than an effect of the implant itself [20]. The absence of differences at 10 years further supports this interpretation, suggesting that early postoperative disparities reflect baseline psychological profiles and early recovery trajectories, which converge over time as longer-term mental wellbeing becomes increasingly influenced by aging and broader psychosocial factors [21]. Registry-based literature cautions that comorbidity prevalence and patient health status often change over time, demonstrated by an increase in comorbidity rates and rising prevalence of specific conditions (e.g., diabetes and renal disease) over recent decades in THA populations [22]. The higher proportion of ASA 3 patients within the Rimfit cohort aligns with documented temporal trends in arthroplasty populations [23], with the comorbidity differences between our cohorts likely reflecting evolving patient health profiles [22, 23]. The relatively small sample of poor OHS scores (< 27) investigated revealed no difference between the cohorts other than the increased proportion of ASA 2 in the Rimfit cohort, reaffirming no stark differences between cohorts other than comorbidities.

### Linear regression preoperative PROMs

Funding source, as well as female sex and BMI, appear to consistently impact preoperative outcomes. Publicly funded patients showed worse preoperative scores, likely due to longer wait times [24], limited supportive care, higher comorbidities, barriers to health literacy, unemployment [25], and systemic inequities that result in patients needing greater disease severity to become eligible for surgery [26]. Female sex has frequently been associated with poorer pre- and postoperative PROMs in THA. Females tend to present for surgery with greater pain, functional limitations, and comorbidity burden [27], which may partly reflect differences in pain perceptions, bone morphology, and delayed access to surgery [28]. Alongside these factors, higher rates of obesity and osteoporosis can influence surgery [29], recovery, and perceived outcomes [30]. Females need to be considered a priority in future risk stratification models [31] with female-specific management potentially beneficial [30].

Body mass index as a predictor of poorer preoperative PROMs in both cohorts, and higher ASA ratings in the Rimfit cohort, also suggests that comorbidity burden may significantly impact recovery trajectories, aligning with findings that a higher number of comorbidities introduces a higher risk of postoperative complications. Several studies [23, 32, 33] highlighted the value of documenting comorbidities in greater detail. While indices such as ASA correlate with THA outcomes, they provide limited guidance for individualised treatment. Podmore et al. [32] noted that specific conditions influence recovery differently, and Jämsen et al. [33] found that implant survival hazard ratios are affected by cardiac, endocrine, oncologic, and mental health conditions in both univariate and multivariate analysis. It may be prudent for regional registries like the one analysed here to record more detailed comorbidity data and to pursue future comorbidity specific THA research to determine which comorbidities may predict the poorest outcomes.

Preoperatively, Māori presented significant and clinically meaningful poorer preoperative VR-12 mental sores, indicating ethnic disparity in health outcomes even before surgery. Such disparities could be related to psychosocial, cultural, and systemic factors [34]. Results must be interpreted with caution, however, given the limited number of NZ Māori in the ECF group (*n* = 9) restraining our ability to robustly analyse preoperative PROMs.

### Linear regression postoperative PROMs

In both cohorts, preoperative PROMs consistently demonstrated the largest predictive contribution to postoperative outcomes, consistent with previous studies [8, 35, 36]. Outside of preoperative scores, age at surgery influenced both cohorts while NZ Māori ethnicity predicted poorer physical postoperative outcomes in the Rimfit cohort, and ASA 3 comorbidity status in the ECF cohort. The limited number of Māori in our study hinders the robustness of analyses, limits regression modelling, and precludes assessment of ethnicity as a true, independent predictor, highlighting Māori underrepresentation in THA surgery. In our study, Māori represented < 5% of the cohort, a clear underrepresentation considering Māori represent 30.6% of the population in the Bay of Plenty region [37]. Contributing factors for Māori under representation may include structural (colonisation, governance, socioeconomic) and intermediary (education, work, accessibility, lifestyle) determinants, highlighting the need to address upstream systemic and cultural barriers, not just surgical implants [38, 39].

Whether preoperatively or postoperatively, all patient factors investigated influenced patient outcomes to a certain extent. Although our results provide some insight, the low coefficient of determination of our models (R^2^ ~ 0.34 at best) indicates that other potential influencers may affect scores more readily or assist in explaining a greater proportion of PROM variance, such as smoking status [40], activity levels [41], postoperative rehabilitation, health literacy, education, and care [42]. Considering the ECF implant is no longer used in NZ, the outcomes of the Rimfit linear regression models may be more relevant to future comparative studies with other specific implants.

The rationale for examining 10-year PROMs was to assess whether incremental advances in polyethylene design translate into sustained patient-perceived benefit over the longer term. Given the similarity in implant geometry and fixation, large early differences were not anticipated; rather, any potential differences were hypothesised to emerge with longer follow-up, where polyethylene durability may plausibly influence pain, function, or activity [43]. While radiographic outcomes and implant survivorship are critical measures of implant performance, PROMs capture the patient experience of that performance. The absence of clinically meaningful differences in 10-year PROMs therefore suggests that both designs provide durable and comparable PROMs, complementing existing survivorship data.

## Conclusion

From a patient outcomes perspective, the Rimfit cup shows no significant difference in PROMs to the ECF at 10 years, with both implants demonstrating sustained improvements in PROMs and no statistically significant differences in physical function outcomes over 10 years. The Rimfit cohort presented with superior VR-12 mental scores preoperatively, sustained at 1-year, and at 5-year suggesting baseline differences in mental wellbeing. Socioeconomic status, sex, and comorbidity status (i.e., ASA) were identified as significant clinical predictors of poorer preoperative physical outcomes in both cohorts. For the Rimfit, Māori ethnicity was a predictor of poorer preoperative mental health and postoperative physical PROM scores. Postoperatively, older age at surgery was detrimental to PROMs outcomes in both cohorts. Findings should be interpreted within the temporal and health-system context in which each implant was used.

## Limitations

Our analysis included patients with at least one completed PROM, representing 1360 of 1630 (84%) total THA recipients. Patients without any PROMs may differ systematically from responders, for example in baseline health, engagement with care, or postoperative outcomes. While the proportion of missing PROMs was relatively small and similar across groups, this selection may introduce bias and limit generalisability of our findings to the full cohort.

As with all registry-based analyses, this study is subject to selection bias. Inclusion was limited to patients captured by the registry and to those with at least one completed PROM, which may preferentially include more engaged or higher-functioning patients and lead to overestimation of postoperative outcomes. Differences in funding status and temporal changes in referral patterns, surgical practice, and PROM collection may also influence baseline characteristics and observed outcomes independent of implant choice. These factors should be considered when interpreting the findings, and causal inferences should be made with caution. While the large sample size improves precision and statistical power, it does not eliminate the possibility of bias or residual confounding.

The width of follow-up windows reflects the pragmatic nature of registry-based PROM collection, where follow-up timing is not fixed but occurs within predefined intervals. To minimise potential bias, 1-year follow-up was selected as the primary analytical endpoint due to the largest sample size, greatest temporal clustering of responses and lack of statistically significant differences observed in mean PROMs across 1-, 5-, and 10-year follow-up, suggesting that variation in follow-up timing did not meaningfully influence comparative conclusions.

## Supplementary Information

Below is the link to the electronic supplementary material.

Supplementary Material 1

Supplementary Material 2

## Data Availability

Accessibility of protocol, raw data, and programming code can be obtained from the authors by request.

## References

[1] Evans JT, Evans JP, Walker RW et al (2019) How long does a hip replacement last? A systematic review and meta-analysis of case series and national registry reports with more than 15 years of follow-up. Lancet 393(10172):647–654. 10.1016/S0140-6736(18)31665-9 30782340 10.1016/S0140-6736(18)31665-9 PMC6376618

[2] Pearce A, Butcher A, Hebert-Losier K (2025) Outcomes of cemented and hybrid primary total hip arthroplasty for osteoarthritis: a systematic review with narrative synthesis. Arch Orthop Trauma Surg 145(1):388. 10.1007/s00402-025-06007-3 40719919 10.1007/s00402-025-06007-3 PMC12304010

[3] Yamate S, Hamai S, Lyman S et al (2023) Clinical evaluation of hip joint diseases: total hip arthroplasty to support patients’ quality of life. J Joint Surg Res 1(1):18–25. 10.1016/J.JJOISR.2022.12.004

[4] Quiceno E, Correa CD, Tamayo JA et al (2024) Statistical models and implant customization in hip arthroplasty: seeking patient satisfaction through design. Heliyon 10(20):e38832. 10.1016/j.heliyon.2024.e38832 39506933 10.1016/j.heliyon.2024.e38832 PMC11538734

[5] Langlois J, Hamadouche M (2023) What have we learned from 20 years of using highly crosslinked PE in total hip arthroplasty? Orthop Traumatol Surg Res 109(1S):103457. 10.1016/j.otsr.2022.103457 36302450 10.1016/j.otsr.2022.103457

[6] Kalairajah Y, Azurza K, Hulme C et al (2005) Health outcome measures in the evaluation of total hip arthroplasties: a comparison between the Harris hip score and the Oxford hip score. J Arthroplasty 20(8):1037–1041. 10.1016/j.arth.2005.04.017 10.1016/j.arth.2005.04.017 16376260

[7] Maggs JL, Smeatham A, Whitehouse SL et al (2016) The exeter contemporary flanged cemented acetabular component in primary total hip arthroplasty. Bone Joint J 98B(3):307–312. 10.1302/0301-620X.98B3.35901 10.1302/0301-620X.98B3.35901 26920954

[8] Pearce A, Joshi C, Chan G et al (2025) Fifteen-year patient-reported outcomes of a cemented flanged cup and stem combination in primary total hip arthroplasty: a New Zealand study. HIP Int. 10.1177/11207000251371132 41169163 10.1177/11207000251371132 PMC12876424

[9] Boyle AB, Zhu M, Frampton C et al (2023) Comparing modern uncemented, hybrid and cemented implant combinations in older patients undergoing primary total hip arthroplasty. A New Zealand joint registry study. Arch Orthop Trauma Surg 143(6):3597–3604. 10.1007/s00402-022-04610-2 36102955 10.1007/s00402-022-04610-2

[10] Pearce A, Joshi C, Chan G et al (2025) 10-Year survival comparison of two cemented implants in primary total hip arthroplasty for osteoarthritis—a New Zealand regional study. Arch Orthop Trauma Surg. 10.1007/s00402-025-06092-4 41165855 10.1007/s00402-025-06092-4 PMC12575574

[11] vanWinterswijk PJTS, Whitehouse SL, Timperley AJ et al (2017) The rim cutter does not show an advantage over modern cementing techniques: a five-year radiological and clinical follow-up of the rim cutter used with flanged acetabular components. Bone Joint J 99–B(11):1450–1457. 10.1302/0301-620X.99B11.BJJ-2017-0138.R1 10.1302/0301-620X.99B11.BJJ-2017-0138.R1 29092983

[12] Kalairajah Y, Azurza K, Hulme C et al (2005) Health outcome measures in the evaluation of total hip arthroplasties. A comparison between the Harris Hip Score and the Oxford Hip Score. J Arthroplasty 20(8):1037–1041. 10.1016/J.ARTH.2005.04.017 16376260 10.1016/j.arth.2005.04.017

[13] Posit T (2023) RStudio: integrated development environment for R. Posit software. PBC, Boston, MA

[14] von Elm E, Altman DG, Egger M et al (2007) The strengthening the reporting of observational studies in epidemiology (STROBE) statement: guidelines for reporting observational studies. PLoS Med 4(10):e296. 10.1371/journal.pmed.0040296 17941714 10.1371/journal.pmed.0040296 PMC2020495

[15] Molino J, Harrington J, Racine-Avila J et al (2022) Deconstructing the minimum clinically important difference (MCID). Orthop Res Rev 14:35–42. 10.2147/ORR.S349268 35210873 10.2147/ORR.S349268 PMC8860454

[16] Deckey DG, Verhey JT, Christopher ZK et al (2023) Discordance abounds in minimum clinically important differences in THA: a systematic review. Clin Orthop Relat Res 481(4):702–714. 10.1097/CORR.0000000000002434 36398323 10.1097/CORR.0000000000002434 PMC10013655

[17] Beard DJ, Harris K, Dawson J et al (2015) Meaningful changes for the Oxford hip and knee scores after joint replacement surgery. J Clin Epidemiol 68(1):73. 10.1016/J.JCLINEPI.2014.08.009 25441700 10.1016/j.jclinepi.2014.08.009 PMC4270450

[18] Langenberger B, Schrednitzki D, Halder AM et al (2023) Predicting whether patients will achieve minimal clinically important differences following hip or knee arthroplasty. Bone Joint Res 12(9):512–521. 10.1302/2046-3758.129.BJR-2023-0070.R2 37652447 10.1302/2046-3758.129.BJR-2023-0070.R2 PMC10471446

[19] Yeo MGH, Goh GS, Chen JY et al (2020) Are Oxford hip score and western Ontario and McMaster Universities Osteoarthritis Index useful predictors of clinical meaningful improvement and satisfaction after total hip arthroplasty? J Arthroplasty 35(9):2458–2464. 10.1016/j.arth.2020.04.034 32416955 10.1016/j.arth.2020.04.034

[20] Aspari AR, Lakshman K (2018) Effects of pre-operative psychological status on post-operative recovery: a prospective study. World J Surg 42(1):12–18. 10.1007/s00268-017-4169-2 28795208 10.1007/s00268-017-4169-2

[21] O’Connor JP, Holden P, Gagnier JJ (2022) Systematic review: preoperative psychological factors and total hip arthroplasty outcomes. J Orthop Surg Res 17(1):457. 10.1186/s13018-022-03355-3 36253795 10.1186/s13018-022-03355-3 PMC9575292

[22] Penfold CM, Judge A, Sayers A et al (2022) Temporal trends in comorbidity in adult elective hip and knee arthroplasty patients in England: a national population-based cohort study from the National Joint Registry. Bone Joint J 104–B(9):1052–1059. 10.1302/0301-620X.104B9.BJJ-2022-0013.R1 36047019 10.1302/0301-620X.104B9.BJJ-2022-0013.R1

[23] Reisinger L, Cozowicz C, Poeran J et al (2025) Trends in comorbidities and complications among patients undergoing elective total hip and knee arthroplasty in the USA. Anaesthesia 80(5):543–550. 10.1111/anae.16529 39756811 10.1111/anae.16529

[24] Karayiannis PN, Warnock M, Cassidy R et al (2023) The painful truth of waiting for hip and knee arthroplasty in Northern Ireland: a cohort study of the worst NHS waiting lists for elective hip and knee arthroplasty. Bone Joint 105–B(7):783–794. 10.1302/0301-620X.105B7.BJJ-2023-0078.R1 10.1302/0301-620X.105B7.BJJ-2023-0078.R1 37399093

[25] Hoelen TA, Schotanus M, van Kuijk S et al (2023) The relation between socioeconomic status and patient symptoms before and one year after lower extremity arthroplasty. J Orthop 39:11–17. 10.1016/j.jor.2023.03.011 37089622 10.1016/j.jor.2023.03.011 PMC10120353

[26] Alvarez P, McKeon J, Spitzer A et al (2022) Socioeconomic factors affecting outcomes in total knee and hip arthroplasty: a systematic review on healthcare disparities. Arthroplasty 4(1):36. 10.1186/s42836-022-00137-4 36184658 10.1186/s42836-022-00137-4 PMC9528115

[27] Zhang B, Rao S, Mekkawy KL et al (2023) Risk factors for pain after total hip arthroplasty: a systematic review. Arthroplasty 5(1):19. 10.1186/s42836-023-00172-9 37009894 10.1186/s42836-023-00172-9 PMC10069042

[28] Jüni P, Low N, Reichenbach S et al (2010) Gender inequity in the provision of care for hip disease: population-based cross-sectional study. Osteoarthr Cartil 18(5):640–645. 10.1016/J.JOCA.2009.12.010 10.1016/j.joca.2009.12.010 20167302

[29] Grant S, Pincus D, Ruangsomboon P et al (2024) Sex differences in complications following total hip arthroplasty: apopulation-based study. J Arthroplasty 39(12):3004–3008. 10.1016/j.arth.2024.05.062 38797453 10.1016/j.arth.2024.05.062

[30] Solarino G, Bizzoca D, Moretti AM et al (2022) Sex and gender-related differences in the outcome of total hip arthroplasty: a current concepts review. Medicina (Kaunas) 58(12):1702. 10.3390/medicina58121702 10.3390/medicina58121702 PMC9788147 36556904

[31] Patel AP, Gronbeck C, Chambers M et al (2020) Gender and total joint arthroplasty: variable outcomes by procedure type. Arthroplast Today 6(3):517–520. 10.1016/j.artd.2020.06.012 32743033 10.1016/j.artd.2020.06.012 PMC7387670

[32] Podmore B, Hutchings A, van der Meulen J et al (2018) Impact of comorbid conditions on outcomes of hip and knee replacement surgery: a systematic review and meta-analysis. BMJ Open 8(7):e021784. 10.1136/bmjopen-2018-021784 29997141 10.1136/bmjopen-2018-021784 PMC6082478

[33] Jämsen E, Peltola M, Eskelinen A et al (2013) Comorbid diseases as predictors of survival of primary total hip and knee replacements: a nationwide register-based study of 96 754 operations on patients with primary osteoarthritis. Ann Rheum Dis 72(12):1975–1982. 10.1136/annrheumdis-2012-202064 23253916 10.1136/annrheumdis-2012-202064 PMC3841739

[34] Loring B, Paine SJ, Robson B, Reid P (2022) Analysis of deprivation distribution in New Zealand by ethnicity, 1991–2013. N Z Med J 135:31–40 36356267 10.26635/6965.5879

[35] Appiah KOB, Khunti K, Kelly BM et al (2023) Patient-rated satisfaction and improvement following hip and knee replacements: development of prediction models. J Eval Clin Pract 29(2):300–311. 10.1111/jep.13767 36172971 10.1111/jep.13767

[36] Orr MN, Klika AK, Emara AK et al (2022) Dissatisfaction after total hip arthroplasty associated with preoperative patient-preported outcome phenotypes. J Arthroplasty 37(7S):S498–S509. 10.1016/j.arth.2022.02.042 35279339 10.1016/j.arth.2022.02.042

[37] Stats-NZ Our region: Bay of Plenty. https://www.stats.govt.nz/infographics/detailed-regional-infographics-from-2023-census/our-region-bay-of-plenty/#:~:text=Under%2015%20years:%2019.9%25%20%7C,to%20talk):%202.1%25%20%7C%206%2C948. Accessed 28 June 2024

[38] Rahiri JL, Alexander Z, Harwood M et al (2018) Systematic review of disparities in surgical care for Maori in New Zealand. ANZ J Surg 88(7–8):683–689. 10.1111/ans.14310 29150888 10.1111/ans.14310

[39] Gleeson C, Zhu M, Frampton C, Young S, Poutawera V, Mutu-Grigg J (2023) Ethnic differences in the utilization and outcome of patients undergoing total hip and knee arthroplasty in New Zealand. Bone Joint 105:51. 10.1302/1358-992X.2023.2.051

[40] Yue C, Cui G, Ma M et al (2022) Associations between smoking and clinical outcomes after total hip and knee arthroplasty: a systematic review and meta-analysis. Front Surg 9:1–18. 10.3389/fsurg.2022.970537 10.3389/fsurg.2022.970537 PMC9666709 36406352

[41] Reine ST, Xi Y, Chhabra A et al (2022) Does preoperative activity level affect postoperative outcomes following total hip arthroplasty? J Arthroplasty 37(7):1314–1319. 10.1016/j.arth.2022.03.009 35276277 10.1016/j.arth.2022.03.009

[42] Nicolau C, Mendes L, Ciriaco M et al (2022) Educational intervention in rehabilitation to improve functional capacity after hip arthroplasty: a scoping review. J Pers Med 12(5):656. 10.3390/jpm12050656 10.3390/jpm12050656 PMC9147380 35629079

[43] Ronda J, Wojnarowski P (2013) Analysis of wear of polyethylene hip joint cup related to its positioning in patient’s body. Acta Bioeng Biomech 15(1):77–86 23957232

