# Supplementary material for: Patient-reported outcome measure comparison of two cemented primary total hip arthroplasty implant combinations for osteoarthritis: a regional New Zealand study
Source: Eur J Orthop Surg Traumatol. 2026 Apr 2;36(1):154. doi: 10.1007/s00590-026-04734-w (PMC13046578; doi:10.1007/s00590-026-04734-w)
Supplement: Supplementary file 1 — Supplementary Material 1 [file 590_2026_4734_MOESM1_ESM.docx]

Supplementary Table 1. Poor Oxford Hip score (<27) analysis

|  | **Rimfit** | **ECF** | *p*-value |
| --- | --- | --- | --- |
| Total | 53 | 7 | 0.134 |
| Age (mean ±SD | 71.6 ±8.3 | 74.9 ±10.1 | 0.566 |
| BMI (mean ± SD) | 29.3 ±4.7 | 26.6 ± 3.6 | 0.106 |
| Sex, n |  |  |  |
| Male | 20 | 3 | 1.00 |
| Female | 33 | 4 |  |
| ASA, n |  |  |  |
| 1 | 3 | 1 | 0.217 |
| 2 | 26 | 2 |  |
| 3 & 4 | 15 | 4 |  |
| Missing† |  |  |  |
| Funding, n |  |  |  |
| Public | 29 | 4 | 0.686 |
| Private | 15 | 3 |  |
|  |  |  |  |
| Ethnicity, n |  |  |  |
| NZEU | 44 | 7 | 0.580 |
| Māori | 9 | 0 |  |
| Abbreviations: ASA, American Society of Anesthesiologists grading; BMI, body mass index; NZEU, New Zealand European  † Missing values have not been included in proportion difference calculations  * Significance set at *p*<0.05 | | | |
