# Supplementary material for: Patient-reported outcome measure comparison of two cemented primary total hip arthroplasty implant combinations for osteoarthritis: a regional New Zealand study
Source: Eur J Orthop Surg Traumatol. 2026 Apr 2;36(1):154. doi: 10.1007/s00590-026-04734-w (PMC13046578; doi:10.1007/s00590-026-04734-w)
Supplement: Supplementary file 2 — Supplementary Material 2 [file 590_2026_4734_MOESM2_ESM.pdf]

Supplementary Table 1. Poor Oxford Hip score (<27) analysis

	**Rimfit**	**ECF**	*p* - value
Total	53	7	0.134
Age (mean ±SD)	71.6 ±8.3	74.9 ±10.1	0.566
BMI (mean ± SD)	29.3 ±4.7	26.6 ± 3.6	0.106
Sex, n			
Male	20	3	1.00
Female	33	4	
ASA, n			
1	3	1	0.217
2	26	2	
3 & 4	15	4	
Missing†	9		
Funding, n			
Public	29	4	0.686
Private	14	3	
Ethnicity, n			
NZEU	44	7	0.580
Māori	9	0	

Abbreviations: ASA, American Society of Anesthesiologists grading; BMI, body mass index; NZEU, New Zealand European

† Missing values have not been included in proportion difference calculations

* Significance set at p<0.05

Supplementary Table 2. Multiple linear regression results for pre-operative patient-reported outcomes

**Exeter X3 Rimfit**									
Predictor	Sample (n)	R^2	*F-statistic*	*DF*	Estimate (β)	*SE*	*t*-value	*p*-value	Interpretation
**Oxford pre-op (0-48)**	767	0.31	67.07	5, 762				<0.001*	
(intercept)					30.91	1.61	19.23	<0.001*	Baseline: Female, private funding, ASA I
BMI					-0.27	0.06	-4.73	<0.001*	↑ BMI → ↓ Oxford score
Sex (Male)					1.98	0.57	3.53	<0.001*	Males score ~ 2 points better than females
Funding (Public)					-8.25	0.58	-14.28	<0.001*	Public patients score ~ 8 pts worse than private
ASA (2)					-2.10	0.80	-2.64	0.009*	ASA 2 score ~ 2 points worse than ASA 1
ASA (3)					-3.50	0.94	-3.70	<0.001*	ASA 3 score ~ 3.5 points worse than ASA 1
**WOMAC total pre-op (0-96)**	758	0.29	60.45	5, 753				<0.001*	
(intercept)					28.71	3.38	8.47	<0.001*	Baseline: Female, private funding, ASA (1)
BMI					0.52	0.12	4.36	<0.001*	↑ BMI → ↑ (worse) WOMAC
Sex (Male)					-3.98	1.18	-3.37	<0.001*	Females score ~ 4 points worse than males
Funding (Public)					16.25	1.22	13.35	<0.001*	Public patients score ~ 16 points worse than private
ASA (2)					5.10	1.66	3.06	0.002*	~ 5 points worse than ASA (1)
ASA (3)					7.85	1.99	3.96	<0.001*	~ 8 points worse than ASA (1)
**VR-12 physical pre-op ($\bar{x}$ =50)**	763	0.20	38.37	5, 764				<0.001*	
(intercept)					38.08	1.60	23.74	<0.001*	Baseline: Female, private funding, ASA (1)
BMI					-0.20	0.06	-3.64	<0.001*	↑ BMI → ↓ physical HRQOL
Sex (Male)					2.05	0.56	3.64	<0.001*	Males score 2 points better physical HRQOL than female
Funding (Public)					-5.56	0.58	-9.64	<0.001*	Public funding ~5.5 points worse physical HRQOL to private
ASA (2)					-3.56	0.79	-4.50	<0.001*	~3.5 points worse physical HRQOL than ASA (1)
ASA (3)					-4.21	0.94	-4.47	<0.001*	~4 points worse physical HRQOL than ASA (1)
**VR-12 mental pre-op ($\bar{x}$ = 50)**	824	0.15	29.16	5, 819				<0.001*	
(intercept)					52.28	1.11	47.00	<0.001*	Baseline: Female, private funding, non-Māori, ASA (1)
Sex (Male)					2.79	0.86	3.25	0.001*	Males score ~ 3 points better mental HRQOL than females
Funding (Public)					-6.80	0.88	-7.76	<0.001*	Public funding ~7 points poorer mental HRQOL to private
ASA (2)					-3.30	1.22	-2.70	0.007*	~ 3 points worse mental HRQOL than ASA (1)
ASA (3)					-6.48	1.42	-4.56	<0.001*	~ 6.5 points worse mental HRQOL than ASA (1)
Ethnicity (Māori)					-6.25	1.82	-3.44	<0.001*	Māori score ~ 6 points worse mental HRQOL

Exeter Contemporary Flanged cup

Predictor	Sample (n)	R^2	*F-Statistic*	*DF*	Estimate (β)	*STD Error*	*t*-value	P-value	Interpretation
**Oxford pre-op (0-48)**	263	0.33	36.91	3, 222				<0.001*	
(intercept)					26.91	2.94	9.16	<0.001*	Baseline: Female, private funding
BMI					-0.25	0.10	-2.44	0.015*	↑ BMI → ↓ Oxford score
Sex (Men)					3.20	0.98	3.28	0.001*	Males score ~ 3 points better than females
Funding (Public)					-10.46	1.10	-9.56	<0.001*	Public patients score ~ 10 pts worse than private
**WOMAC total pre-op (0-96)**	262	0.34	30.68	3, 172				<0.001*	
(intercept)					22.55	7.80	2.89	0.004*	Baseline: Female, private funding
BMI					0.95	0.28	3.42	<0.001*	↑ BMI → ↑ (worse) WOMAC
Sex (Men)					-7.80	2.45	-3.19	0.002*	Females score ~ 8 points worse than males
Funding (Public)					22.85	2.81	8.12	<0.001*	Public patients score ~ 23 points worse than private
**VR12 physical pre-op ($\bar{x}$ = 50)**	263	0.12	12.28	2, 177				<0.001*	
(intercept)					27.30	0.88	31.20	<0.001*	Baseline: Female, private funding
Funding (Public)					-5.82	1.33	-4.39	<0.001*	Public funding ~6 points worse physical HRQOL to private
Sex (Men)					2.92	1.19	2.46	0.015*	Males score ~6 points better physical HRQOL than female
**VR12 mental pre-op ($\bar{x}$ = 50)**	263	0.30	37.69	2, 177				<0.001*	
(intercept)					47.18	1.18	40.02	<0.001*	Baseline: Female, private funding
Sex (Men)					5.50	1.60	3.44	<0.001*	Males score 5.5 points better mental HRQOL
Funding (Public)					-14.48	1.79	-8.09	<0.001*	Public funding linked to ~14 points poorer mental HRQOL

Abbreviations: ASA, American Society of Anesthesiologists physical status classification; BMI, body mass index; DF, degrees of freedom; F-Stat, F-statistic; HRQOL, health related quality of life; Pre-op, pre-operative; STD, standard; WOMAC, Western Ontario and McMaster Universities; Arthritis Index; VR-12, Veterans Rand 12-item health survey.

* Significance set at $p< 0.05$

Supplementary Table 3. Multiple linear regression results for post-operative patient reported outcomes

Exeter X3 Rimfit cup									
Predictor	Sample (n)	R^2	*F statistic*	*DF*	Estimate (β)	Std. Error	*t*-value	*P*-value	Interpretation
**Oxford post-op (0-48)**	607	0.08	18.55	3, 604				<0.001 *	
Intercept					44.77	2.671	16.76	<0.001*	Baseline: Ethnicity (NZEU)
Oxford pre-op					0.181	0.034	5.36	<0.001*	↑ pre-op score → higher post-op Oxford → better post-op outcome
Age at surgery					-0.093	0.035	-2.63	0.009*	↑ age → lower post-op Oxford → worse post-op outcome
Ethnicity (Māori)					-4.925	1.441	-3.42	<0.001*	NZ Māori score ~5 points lower→ worse post-op outcome
**WOMAC post-op (0-96)**	585	0.08	18	3, 582				<0.001 *	
Intercept					-13.14	5.032	–2.61	0.009*	Baseline: Ethnicity (NZEU)
WOMAC pre-op					0.18	0.03	5.63	<0.001*	↑ Pre-op WOMAC → ↑ post-op WOMAC → worse post-op outcome
Age at surgery					0.24	0.07	3.52	<0.001*	Each 1 year ↑ is a 0.24 ↑ post-op WOMAC → worse post-op outcome
Ethnicity (Māori)					5.83	2.69	2.17	0.031*	NZ Māori score ~ 6 points higher → worse post-op outcome
**VR-12 physical post-op ($\bar{x}$ = 50)**	612	0.11	27.14	3, 609				<0.001 *	
Intercept					58.06	3.85	15.07	< 0.001 *	Baseline: Ethnicity (NZEU)
VR-12 physical pre-op					0.23	0.05	4.75	< 0.001 *	↑ Pre-op VR12 Physical → ↑ post-op VR12 Physical→ better post-op outcome
Age at surgery					-0.27	0.05	-5.65	< 0.001 *	Each 1-year ↑ is a 0.27 ↓ in post-op → worse post-op outcome
Ethnicity (Māori)					–7.56	1.88	-4.03	< 0.001 *	Māori associated with ~8 point lower post-op → worse post-op outcome
**VR-12 mental post-op ($\bar{x}$= 50)**	563	0.14	45.76	2, 561				<0.001 *	
Intercept					46.54	2.77	16.83	< 0.001 *	Baseline
VR12 mental pre-op					0.26	0.03	8.73	< 0.001 *	Each 1-point ↑ pre-op → 0.26-point ↑ post-op → better post-op outcome
BMI					-0.19	0.08	-2.48	0.014*	↓ BMI → 0.19 point ↑ post-op → better post-op outcome

Exeter Contemporary Flanged									
Predictor	Sample (n)	R^2	*F statistic*	*DF*	Estimate	Std. Error	*t*-value	*P*-value	Interpretation
**Oxford post-op (0-48)**	238	0.07	16.07	1, 202				<0.001*	
(intercept)					36.81	1.12	32.84	<0.001*	Baseline
Oxford pre-op					0.22	0.05	4.01	<0.001*	Each 1-point ↑ pre-op → 0.22-point ↑ post-op → better post-op outcome
**WOMAC post-op (0-96)**	235	0.15	10.36	3, 172				<0.001*	

(intercept)					3.29	2.74	1.20	0.232	Baseline: ASA 1
WOMAC pre-op					-0.70	2.08	-0.337	<0.001*	Each 1-point ↑ pre-op → 0.22-point ↑ post-op → poorer post-op outcome
ASA (2)					-1.11	2.04	-0.546	0.586	no significant difference compared to ASA 1
ASA (3)					7.66	2.85	2.69	0.008*	ASA 3 ↓ post-op scores than ASA 1 by ~7.7 points → poorer post-op outcome
**VR12 physical post-op ($\bar{x}$ = 50)**	240	0.28	17.34	4, 175				<0.001*	
(intercept)					58.54	6.66	8.78	<0.001*	Baseline: ASA 1
VR12 physical pre-op					0.53	0.08	6.23	<0.001*	↑ pre-op physical scores ↑ post-op → better post-op outcome
Age at surgery					-0.40	0.09	-4.31	<0.001*	Each year ↑ is a 0.4 ↓ in post-op → worse post-op outcome
ASA (2)					-0.66	1.82	-0.36	0.717	no significant difference vs. ASA 1.
ASA (3)					-5.56	2.52	-2.21	0.029*	ASA 3 ↓post-op scores than ASA1 by ~5.6 points → worse post-op outcome
**VR12 mental post-op ($\bar{x}$ = 50**)	240	0.11	22.29	1, 178				<0.001*	
(intercept)					40.77	2.48	16.46	<0.001*	Baseline
VR12 mental pre-op					0.25	0.05	4.72	<0.001*	higher pre-op mental health scores predict better post-op mental scores.

Abbreviations: ASA, American Society of Anesthesiologists physical status classification; DF, Degrees of freedom; F-Stat, F-statistic; Pre-op, pre-operative; Post-op, post-operative; PROMs, Patient reported outcome measures; STD, Standard; WOMAC, Western Ontario and McMaster Universities Arthritis Index; VR- 12, Veterans Rand 12-item health survey.

* Significance is set at p< 0.05
